# Condensed Tannins as Antioxidants in Ruminants—Effectiveness and Action Mechanisms to Improve Animal Antioxidant Status and Oxidative Stability of Products

**DOI:** 10.3390/ani11113243

**Published:** 2021-11-13

**Authors:** David Soldado, Rui J. B. Bessa, Eliana Jerónimo

**Affiliations:** 1 Centro de Biotecnologia Agrícola e Agro-Alimentar do Alentejo (CEBAL), Instituto Politécnico de Beja (IPBeja), 7801-908 Beja, Portugal; david.soldado@cebal.pt; 2 Centro de Investigação Interdisciplinar em Sanidade Animal (CIISA), Avenida da Universidade Técnica, 1300-477 Lisboa, Portugal; rjbbessa@fmv.ulisboa.pt; 3 Faculdade de Medicina Veterinária, Universidade de Lisboa, Avenida da Universidade Técnica, 1300-477 Lisboa, Portugal; 4 MED—Mediterranean Institute for Agriculture, Environment and Development, Centro de Biotecnologia Agrícola e Agro-Alimentar do Alentejo (CEBAL), 7801-908 Beja, Portugal

**Keywords:** condensed tannins, ruminants, oxidative stress, antioxidant effect, mechanism of action, metabolism

## Abstract

**Simple Summary:**

Condensed tannins (CTs) are secondary plant metabolites known for their antinutritional properties but also for their beneficial attributes for animal health and food quality, including antioxidant activity. Condensed tannins sources, such as plants and agro-industrial by-products or extracts prepared from these vegetal materials, have been used in ruminant diets to improve the animal antioxidant status and the oxidative stability of their products. However, this nutritional strategy has shown inconsistent results. Furthermore, unlike other phenolic compounds with low molecular weight, CTs are high molecular weight oligomers and polymers with poor bioavailability, which limit their absorption into circulation and direct antioxidant effect in living animals and post-mortem. Therefore, the action mechanism by which dietary CT exerts an antioxidant effect on ruminants is poorly understood. So, this review briefly presents the chemical structure of tannins, with particular emphasis on CT chemical structure, summarizes several studies focused on the effect of dietary CT sources on ruminants’ antioxidant status and oxidative stability of their products, and discusses the possible action mechanisms by which CT can exert such effects.

**Abstract:**

Condensed tannins (CTs) are widely distributed in plants, and due to their recognized antioxidant activity are considered as possible natural antioxidants for application in ruminant diets. A wide range of CT-rich sources has been tested in ruminant diets, and their effects on animal antioxidant status and oxidative stability of their products are reviewed in the present work. Possible mechanisms underlying the CT antioxidant effects in ruminants are also discussed, and the CT chemical structure is briefly presented. Utilization of CT-rich sources in ruminant feeding can improve the animals’ antioxidant status and oxidative stability of their products. However, the results are still inconsistent. Although poorly understood, the evidence suggests that CTs can induce an antioxidant effect in living animals and in their products through direct and indirect mechanisms, which can occur by an integrated and synergic way involving: (i) absorption of CTs with low molecular weight or metabolites, despite CTs’ poor bioavailability; (ii) antioxidant action on the gastrointestinal tract; and (iii) interaction with other antioxidant agents. Condensed tannins are alternative dietary antioxidants for ruminants, but further studies should be carried out to elucidate the mechanism underlying the antioxidant activity of each CT source to design effective antioxidant strategies based on the use of CTs in ruminant diets.

## 1. Introduction

Livestock species are frequently exposed to oxidative stress, generated by overproduction of free radicals that cannot be handled by the body’s antioxidant defense capacity, resulting in oxidative damage of several vital cell components and deterioration of many physiological functions, such as growth, reproduction, and immunity [1,2]. 

Oxidative damage in cells and tissues results from the action of several reactive molecular species, generally designated as reactive oxygen (ROS) and nitrogen species (RNS), although some non-radical reactive derivatives of oxygen and nitrogen are also included [2] that are able to induce serious injuries in molecules, such as deoxyribonucleic acid (DNA), lipids, proteins and carbohydrates [3]. A normal cellular metabolism generates and eliminates ROS, both processes being closely related, resulting in a very low level of ROS in living organisms [3]. However, this balance can be disturbed under several circumstances [3] and an imbalance between oxidants and antioxidants in favor of the oxidants results in a status known as oxidative stress [4,5].

Several factors can create favorable conditions for the development of oxidative stress in farm animals. Lipid supplementation of ruminants’ diets has been used for a long time to increase their energy density, and more recently to improve the fatty acid profile of ruminant products by inclusion of lipid sources rich in polyunsaturated fatty acids (PUFA) in diets [6,7,8]. However, due to PUFAs’ high susceptibility to oxidation, these nutritional strategies expose the animals to oxidative stress conditions, while enhancing the susceptibility of products to oxidation which limits its quality and acceptability [5]. Nutritional imbalance, feed contamination, and the presence of mycotoxins are other oxidative stress factors [2]. High environmental temperatures are also associated with increased oxidative stress [9], which constitutes a major concern as to the possible impact of expected climate change with alarming temperature increases and more frequent and intense heat waves. In addition to diet and environmental aspects, other factors such as housing condition, animal management, transportation, mechanical injuries, disease and the physiological state of animals can contribute to oxidative imbalance [2,10,11]. These oxidative stress conditions lead to increased production of free radicals above the capacity of the antioxidant system to eliminate them. The excessive free radicals are available to cause oxidative damage to various cell macromolecules, a process by which more free radicals are generated, triggering a chain of oxidative destruction [12]. 

The animals’ antioxidant status is also reflected in the quality of their products, determining a higher susceptibility or resistance to oxidative deterioration. Animal-derived foods are subjected to oxidative reactions, resulting in deterioration of color and development of undesirable odor and flavor, loss of nutrients such as essential fatty acids, vitamins, and other bioactive compounds, and production of compounds harmful to human health, a major cause of the quality loss in food [13,14]. These oxidative reactions are influenced by intrinsic and extrinsic factors that can promote or inhibit them. Processing and storage conditions are the external factors that mostly affect the oxidative stability of foods [14,15]. The lipid content and composition are one of the intrinsic parameters which great influence food’s oxidative stability, with foods containing high amounts of unsaturated fatty acids, such as foods naturally rich or enriched in *n*-3 PUFA, highly susceptible to lipid oxidation [16,17]. Foods with high levels of prooxidants (e.g., hemeproteins, metals such as Fe and Cu) are also particularly prone to oxidation [14]. In contrast, the presence of antioxidants can limit oxidative reactions and improve the shelf-life of foods [15].

When oxidative stress occurs, an adaptive stress response is triggered to minimize the damaging effects of free radicals, increasing the capability of antioxidant systems to eliminate the free radicals [3]. The endogenous antioxidant system—composed by antioxidant enzymes such as catalase (CAT), glutathione peroxidase (GPx) and superoxide dismutase (SOD), and by non-enzymatic antioxidants such as glutathione (GSH), alpha-lipoic acid, Coenzyme Q, ferritin, uric acid and bilirubin—plays crucial roles in maintaining the redox balance in the body [18]. Antioxidant compounds such as vitamins E, C and β-carotene, which are naturally present in greater or lesser amounts in animal feed, are also strongly involved in the mechanisms of protection against free radicals in the living organism and products [5]. However, under certain circumstances, the endogenous antioxidant system and antioxidant compounds naturally present in the feedstock may not be enough to prevent oxidative damage, being fundamental to increase the levels of antioxidant compounds in the diet to ensure animal welfare and productivity, as well as the quality of products [2]. Synthetically manufactured antioxidants are largely used in animal nutrition [19], but to address consumer concerns on safety of these products there has been an increasing interest in the use of natural antioxidants in the diets of various farm animals. 

As plant secondary metabolites, condensed tannins (CTs) are part of the plant chemical defense system against biotic and abiotic stressors [20,21]. Moreover, CTs present several biological activities with benefits for human and animal health and food quality. Antioxidant, anticancer, antidiabetic, anti-inflammatory, anthelmintic, antimicrobial, immunostimulant and cardio- and neuro- and eye-protective are some of the beneficial health properties of CTs [21,22,23,24,25]. Although they are also known for their antinutritional properties, several CT sources has been applied in livestock species diets with neutral and beneficial effects, such as prevention of bloating, control of internal parasites, reduction of methane production, improvement of the digestive utilization of feed proteins, the fatty acid composition of products, or the productive performance in ruminant animals [26,27,28]. Improved gastrointestinal health and growth performance are some of the beneficial effects of dietary CTs reported in monogastric animals [20,29]. 

Probably due to the association of various diseases with oxidative stress, the antioxidant activity has been one of the most studied biological effects of CTs, in both in vitro and in vivo models. The strong antioxidant capacity of CTs is demonstrated in several in vitro assays [30,31,32], and CT extracts have been successfully applied to retard lipid oxidation in foods [33,34]. Moreover, the antioxidant efficacy of CTs has also been verified in human and animal studies, including both ruminants and monogastric farm animals [21,23,24,26,33]. Due to their recognized antioxidant activity and wide distribution among the plant kingdom, CTs are appointed as good natural antioxidant candidates for application in ruminant diets. However, if the antioxidant effect of CTs is fully proven in in vitro studies and when they are directly added to food products, their in vivo and *post-mortem* antioxidant effects when applied in ruminant diets have been contradictory, with doubts remaining as to the effectiveness of this approach, as well as the action mechanisms. So, the main objective of the current review is to critically analyze the available knowledge on the effect of CT sources’ incorporation in ruminant diets on animal antioxidant status and oxidative stability of their products and discuss the CT antioxidant mechanisms in ruminants. Tannins’ chemical structure will also be briefly reviewed.

## 2. Condensed Tannins’ Chemical Structure 

Tannins are a heterogeneous group of phenolic compounds that can be classified into 3 major groups based on their chemical structures: hydrolysable tannins (HTs), condensed tannins (CTs) and phlorotannins (PTs) [20,35]. The HTs and CTs are found in terrestrial plants, while PTs only appears in brown seaweeds [20,35]. Hydrolysable tannins contain a central unit of glucose or other polyols esterified with gallic acid, named gallotannins, or with heaxahydroxydiphenic acid, named ellagitannins [25]. Phlorotannins comprise a distinct set of polymers, structurally like terrestrial tannins, being formed by polymeric chains of phloroglucinol, connected via C-C and/or C-O-C bonds [36]. 

**Figure 1:**
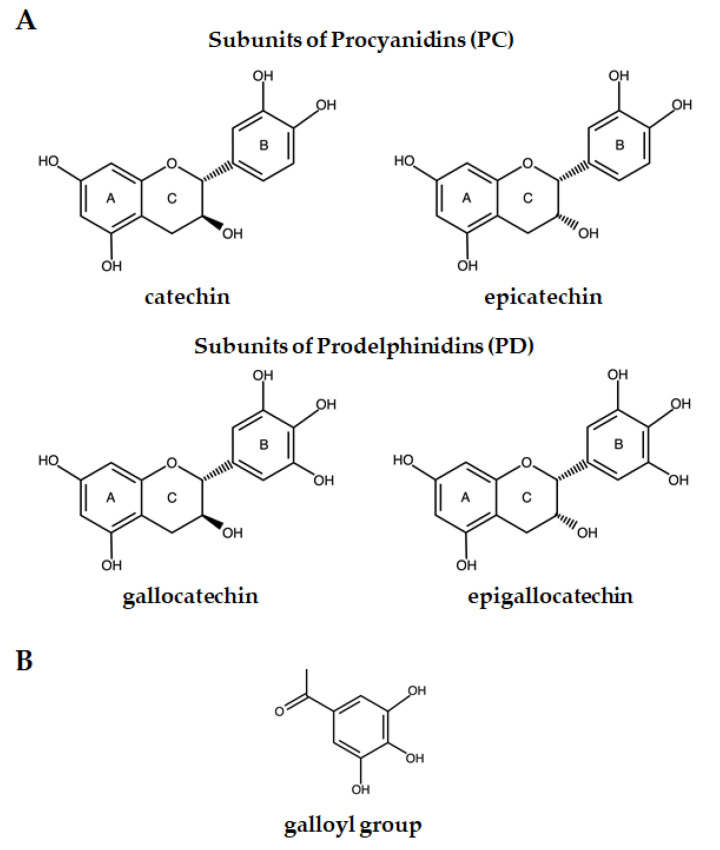
(**A**) Monomeric proanthocyanidins, subunits of procyanidins—catechin and epicatechin and subunits of prodelphinidin—gallocatechin and epigallocatechin; (**B**) galloyl group.

For the purposes of this review, our focus will be on the CTs. Known also as proanthocyanidins, CTs are oligomers and polymers composed of flavan-3-ols linked through C4-C8 and/or C4-C6 bonds, both called B-type CTs, and doubly linked with an additional ether bond at C2 → O → C7 or C2→ O → C5, designed as an A-type CT [23,37]. The size of the CT molecule is characterized by its degree of polymerization, which varies between two and greater than fifty flavan-3-ols subunits [38]. The majority of CTs present in common forage species are mainly composed by four monomeric flavan-3-ol subunits, catechin and epicatechin, gallocatechin and epigallocatechin [39] (Figure 1A), which differ in the number and stereochemistry of hydroxyl groups and the relative stereochemistry of the substituents on the C-ring (i.e., *cis* or *trans*-configuration) [39,40]. The catechin and epicatechin differ from each other only in their stereochemistry of the hydroxyl (OH) group at the C3 carbon on the C-ring. The gallocatechin and epigallocatechin also differ from each other by the spatial orientation of the OH group at C3 on the C-ring [39]. The stereochemistry of the C2 and C3 substituents in the C-ring also differ between flavan-3-ol subunits, in which epicatechin and epigallocatechin show *cis* orientation, whereas catechin and gallocatechin show *trans* orientation [39]. Moreover, catechin and epicatechin have two OH groups on the B-ring, and gallocatechin and epigallocatechin three OH groups on the B-ring [40]. In addition, few plants have CTs containing flavan-3-ol subunits that are modified with galloyl groups at the C3 hydroxyl position (Figure 1B), giving rise to catechin gallate, epicatechin gallate, gallocatechin gallate and epigallocatechin gallate [39]. According to flavan-3-ol subunit composition, CTs can be classified into different subgroups. The two major CT subgroups are procyanidin tannins (PC), composed by catechin and epicatechin and prodelphinidin tannins (PD), which have gallocatechin and epigallocatechin (Figure 1A) [37,39]. The CTs in common forage plants typically contain a complex mixture of PC and PD, while few plants only synthesize PC or PD [41]. 

Taking into account the different pattern of hydroxylation, types of interflavan-3-ol linkages, stereochemistry of the C2 and C3 and degree of polymerization, a great structural diversity of CTs is found in plants [37]. Their structure has relevant influence on the chemistry and biological features of CTs, affecting their absorption and metabolism as well as the protein binding affinity, and the anthelmintic and antioxidant activities, [23,40]. For example, the protein precipitation increases with the CT size [39], and it is reported that the antioxidant activity of CTs is positively related to increase of the polymerization degree [31,42,43,44].

## 3. Condensed Tannins as Antioxidants in Ruminant Diets

A wide range of CT sources has been extensively tested in ruminant diets, as modulators of the ruminal biohydrogenation in order to improve the fatty acid profile of meat and milk fat [26,45], and as natural agents to reduce methane emissions, protect the protein against ruminal degradation, prevent bloating, control internal parasites, and improve animals’ antioxidant status and oxidative stability of their products [20,27,28,39,46,47,48]. The CT sources applied in these nutritional strategies for ruminants include plants (legumes, trees and shrubs) and by-products containing high CT contents or extracts with variable CT purity obtained from these plants and by-products. Despite the large number of CT sources applied in ruminant diets, only a few of them have been tested in terms of their efficacy to improve the animal antioxidant status and limit the oxidative deterioration of products. Standing out are by-products from of wine industry, which are used in their original form (grape pomace) or in the form of extracts (e.g., grape seed extract), by-products from other fruits (carob pulp and peanut skin) and from the forest (pine bark), foliage and extracts from endemic shrubs and trees (e.g., *Cistus ladanifer*, quebracho and mimosa), and widespread legumes forages (sainfoin and sorghum). Plants or by-products rich in CTs may represent a greater proportion of the diet (50–750 g/kg dry matter (DM), Table 1) and provide other nutrients and bioactive compounds in addition to CTs, while CT extracts, which contain high levels of CTs (more than 40% and reaching 95%), are usually incorporated into diets at lower levels (Table 2). So, the antioxidant impact of CT sources applied in ruminant diets will be presented and discussed according to the applied strategy, separating the strategies based in use of CT-rich plants or by-products from CT extracts. 

### 3.1. Condensed Tannin-Rich Plants and Agro-Industrial By-Products

Grape pomace, consisting of grape seeds, skin and pulp, is one of the most studied CT sources in terms of its efficacy to improve ruminants’ antioxidant status and the oxidative stability of their products. Inclusion of 50 and 100 g/kg DM of red wine grape pomace in lamb diets resulted in the improvement of several muscle antioxidant status parameters [49]. A reduction of lipid oxidation, indicated by lower levels of malondialdehyde (MDA), one of the most abundant aldehydes derived from the oxidation of PUFA, and increased total antioxidant capacity and activity of antioxidant enzyme GPx4 in muscle were observed when 100 g/kg DM of grape pomace was incorporated in lamb diets [49]. A lower grape pomace level—50 g/kg DM—was enough to reduce the reactive oxygen species (ROS) content and to increase the SOD activity in muscle [49]. In the same experiment, authors also evaluated the effect of dietary grape pomace inclusion on antioxidant capacity in the testes of ram lambs maintained under housing situations that can lead to increased oxidative stress [50]. The results showed that grape pomace inclusion in diets reduced the ROS and MDA contents in testes of confined lambs to values similar to those found in lambs raised under free-range conditions [50]. Moreover, higher activity of CAT, SOD and GPx4 was also observed in testes of confined lambs that received grape pomace [50]. Inclusion of 50 and 100 g/kg DM of grape pomace in lactating ewes’ diet was also effective in preventing the lipid oxidation and metmyoglobin formation in suckling lamb meat packaged under high oxygen atmospheres (80% of O_2_ and 20% of CO_2_) after 10 days of storage in retail conditions [51]. Conversely, in lamb meat samples also packaged under high O_2_:CO_2_ atmosphere and stored at 2 °C during 14 days, there was no improvement of the lipid and myoglobin stability when 50 g/kg DM of pomace grape was included in the concentrate supplied to the lambs [52]. 

Higher levels of grape pomace in lamb diets than those tested by other authors (50, 100, 150 and 200 g/kg DM) were used in experiments by Chikwanha et al. [53]. The muscle antioxidant activity, evaluated by ferric reducing ability (FRAP) assay decreased over 9 days of storage independently of the grape pomace level in the diet; however, during the first 3 days of storage higher values of antioxidant activity were observed in muscle from lambs fed diets containing 150 and 200 g/kg DM of grape pomace than those fed diets with lower levels of grape pomace. From day 5 to day 9, the difference between the diets was less expressive, but during all storage periods the diet containing of 200 g/kg DM of grape pomace resulted in higher muscle antioxidant activity than a control diet without grape pomace [53]. Consistently, feeding 200 g/kg DM of grape pomace also resulted in the lowest MDA and carbonyl contents in meat from the 5th day of storage [53]. Reduction of the MDA and carbonyl contents and increase of the antioxidant activity (FRAP assay) were also observed in beef when 150 g/kg DM of grape pomace was included in steers diets [54].

In previous studies, the grape pomace was previously dried after incorporation into the diets. Ensiling the grape pomace is an alternative way to conserve this by-product. Inclusion of grape pomace silage (50, 75 and 100 g/kg DM) in the soybean oil-supplemented diet (40 g/kg DM of soybean oil) of dairy cows increased the reducing power of milk to reduce the ferric ions [55]. However, the improvement of antioxidant capacity in milk did not result in reduction of the production of conjugated diene hydroperoxides, a primary product of lipid oxidation [55]. 

Carob pulp is an agro-industrial by-product widely available in the Mediterranean area and commonly used in animal feed [73], which is characterized by its high levels of CTs [74]. However, the effectiveness of carob pulp to improve the oxidative stability of lipids and proteins of lamb meat was not proven when two doses of carob pulp (240 and 350 g/kg) were included in diets [56]. 

Other agro-industrial by-products rich in CTs were also tested in ruminant diets, although the results on their antioxidant effect are still limited. Supplementation of goat diets with pine bark, a by-product of the timber industry containing up to 130 g/kg DM of CTs, also did not affect the meat MDA levels [57]. Meat lipid oxidation was also not affected by inclusion in goat diets of high levels of peanut skin (500 and 750 g/kg), a by-product of the peanut industry [58].

The utilization of endemic species due to their richness in CTs has been another approach to supply CTs in ruminant diets. The aerial part of *Cistus ladanifer*, a perennial shrub very abundant in the Mediterranean area rich in CTs (32–161 g/kg DM of CTs [75]), has been applied in lamb diets with an approach to limit meat lipid oxidation. Incorporation of 250 g/kg DM of *Cistus ladanifer* (leaves and soft stems) in a high-forage diet, supplemented or not with 6% of a blend of soybean and linseed oils (1:2, *v/v*), reduced the MDA levels in meat after lipid oxidation induction [59]. As expected, lipid oxidation increased throughout the 7 days of storage at 2 °C, but lower MDA levels were observed in meats from lambs fed with *Cistus ladanifer*, showing the effectiveness of the dietary *Cistus ladanifer* to enhance the meat’s resistance against lipid oxidation, even in PUFA-enriched meat [59]. Increased resistance against lipid oxidation of lamb meat was confirmed in another work, where increasing levels of *Cistus ladanifer* (50, 100 and 200 g/kg DM) were incorporated in a forage and concentrate diet (1:1) supplemented with 0, 40 and 80 g/kg DM of soybean and linseed oils (1:2, *v/v*) [61]. These results were obtained using a methodology where the lipid oxidation products (MDA) were determined after the lipid oxidation induction [76], not allowing evaluation of the real lipid damage in meat. Nevertheless, the effectiveness of the dietary *Cistus ladanifer* to reduce the lipid oxidation in lamb meat without oxidative induction has also been proven [60,61]. These papers report that, independently of the dietary oil supplementation level, increasing levels of *Cistus ladanifer* (50, 100 and 200 g/kg DM) in diets gradually reduced the MDA levels in meat samples stored for 7 days at 2 °C [61]. Despite that, the muscle antioxidant status evaluated by ferric reducing ability (FRAP assay) and radical scavenging ability (TEAC assay) and the total phenolic content of muscle was unaffected by increasing levels of *Cistus ladanifer* in the diet [61]. In this experiment, all diets were supplemented with the same levels of vitamin E (22.5 mg/kg), and even with the expected prevention of the lipid oxidation in meat by vitamin E, the *Cistus ladanifer* resulted in a significant protection of the meat against lipid oxidation. In a more recent work, the partial replacement of dehydrated lucerne with 150 g/kg DM of *Cistus ladanifer* in oil supplemented diets (50–60 g/kg DM of soybean oil) composed by 1:1 forage and concentrate did not reduced the MDA levels in lamb meat [62]. In this experiment, all diets were also supplemented with vitamin E (22.5 mg/kg), which may have masked a possible beneficial of *Cistus ladanifer* on lipid stability. Differences in basal diets, levels and type of lipid supplement and inclusion of other antioxidant compounds in diets might have created a different antioxidant–pro-oxidant balance among experiments, leading to the inconsistent results on the antioxidant effect of the dietary *Cistus ladanifer* [75]. 

The inclusion of two different CT-rich woody species—*Larrea divaricata* (125 g/kg DM of leaves) and *Acacia aroma* (125 g/kg DM of leaves)—in goat diets composed by forage and concentrate did not change the antioxidant activity (DPPH assay) and total phenols content in meat, but reduced the MDA levels in meat stored at 4 °C for 6 days, at −18 °C for 30 days and at 26 °C for 6 h [63]. Delgadillho-Puga et al. [64] also did not observed differences in milk antioxidant activity evaluated by DPPH assay in dairy goats fed a diet composed of forage:grain concentrate (60:40) containing increasing levels of *Acacia farnesiana* pods (0, 100, 200 and 300 g/kg DM). On the other hand, when the FRAP and oxygen radical absorbance capacity (ORAC) assays were used, an increase of the antioxidant activity of milk was reported, reaching similar or higher values than those presented in the milk of goats fed exclusively on pasture. Increasing levels of total phenols in goats’ milk with increasing amounts of *Acacia farnesiana* pods in diets was also observed; however, the catechin levels remained unchanged. 

The impact of some native species on animal antioxidant status was evaluated by analyses of several antioxidant parameters in the erythrocytes, including enzymatic and non-enzymatic antioxidants and lipid peroxidation parameters. In cows, the dietary replacement of rice bran by dried and ground leaves of *Ficus bengalensis* (119 g/kg in concentrate), a tanniferous native tree from the Indian Subcontinent [65], seems to enhance the animal antioxidant status, increasing the intracellular GSH and the activity of antioxidant enzymes SOD and CAT, while reducing the MDA levels and increasing the level of total thiol (T-SH) groups that act as intracellular antioxidants by scavenging free radicals through enzymatic reactions [65]. Similar results were also verified in lambs, when increasing levels of ground leaves of *Ficus infectoria* (evergreen tree abundant in northern parts of India) were included in their diets (106, 159 and 212 g/kg) [66]. Inclusion of *Ficus infectoria* and *Psidium guajava* leaf mixture (70:30) in concentrate feed (100, 150 and 200 g/kg) supplied to lambs enhanced the erythrocytic antioxidant status, increasing the intracellular activity of SOD and CAT and the levels of GST, GSH and total and protein thiol (P-SH) [67]. Increased levels of GSH and SOD activity in the blood were also observed in goats that received oak leaves (*Quercus electrophori*) containing 3.35% of CT and 3.10% of HT, suggesting an improvement in the antioxidant status of those animals [68]. 

Forage legumes with high levels of CTs, such as sainfoin and high-tannin sorghum, were also evaluated in terms of their effectiveness to limit meat lipid oxidation when incorporated in ruminant diets. Feeding lambs with silages containing sainfoin improved the lipid oxidative stability of meat subjected to pro-oxidant conditions (cooking and incubation with pro-oxidant catalysts) compared to grass silages [69]. Consistently, Lobón et al. [70] reported lower levels of lipid oxidation from day 7 to day 14 of storage at 4 °C in meat of suckling lambs when dams grazed on a sainfoin pasture compared to meat of suckling lambs from dams fed a total mixed ration. The protective effect of high-tannin sorghum against lipid oxidation in meat was contradictory, reducing the MDA levels in vacuum-packaged beef, while increased the lipid oxidation during aerobic display of the beef (14 days at 4 °C) [71]. In this work, the activity of antioxidant enzymes in muscle was not affected by high-tannin sorghum.

Differences in CT chemical structure and concentration in the diet, as well as the diversity of basal diets used and the presence of other bioactive compounds with antioxidant activity in the diet, can help to explain the inconsistent results on the antioxidant effect of CT-rich plants and by-products, either between different CT sources, or with the same CT source. It is also important to note that within each CT source type, the CT levels can be variable among experiments, since the biosynthesis of phenolic compounds, such as CTs, depends on several factors, including variety, plant/fruit development stage, environmental temperature and water availability [21,77,78,79]. Thus, the utilization of the same level of a certain plant or by-product may not correspond to an equal CT amount in the diet. Another important aspect when CTs is provided to animals through the inclusion of plants or by-products in diets is the presence of other antioxidant compounds in addition to CTs in the vegetable material, such as other phenolic compounds and vitamins, which makes it impossible to isolate the contribution of CTs to the antioxidant effect.

### 3.2. Condensed Tannin Extracts 

Utilization of CT extracts allows a better understanding of the specific effect of dietary CTs on ruminant antioxidant status and oxidative stability of their products. Quebracho, mimosa and grape seed have been the main CT extracts applied in ruminant diets, as possible sources of antioxidants (Table 2). 

The protective effect of dietary quebracho CTs against lipid oxidation was verified by Lobón et al. [80] in meat of suckling lambs when dams fed pasture or hay were supplemented with 300 g/head of concentrate containing 10% of quebracho. Conversely, the supplementation of fattening concentrate of the weaned lambs with 50 g/kg of quebracho did not affect the MDA levels in meat over 14 days of storage [70]. No effect of dietary supplementation with quebracho on lamb meat lipid oxidation was also reported by Brogna et al. [81], when including 80 g/kg of quebracho in lamb diets, and by Luciano et al. [82], who supplemented the barley-based concentrate with 89 g/kg DM of quebracho. However, in this last study, Luciano et al. [82] also observed that supplementation of lamb diets with quebracho reduced the metmyoglobin formation and produced an improvement in the muscle antioxidant capacity evaluated using FRAP and TEAC assays and increased the concentration of total phenolic compounds in muscle [83]. Improvement of the liver overall antioxidant status by dietary supplementation with quebracho CT was also observed by López-Andrés et al. [84], who reported higher values of FRAP and total phenolic content in the liver of lambs fed a concentrate containing 64 g/kg of quebracho in contrast to the control ones. In turn, Buccioni et al. [85] found that supplementation of concentrate with 52.8 g/kg DM of quebracho was not able improve the oxidative status in lactating grazing ewes, when they determined the MDA levels in plasma of ewes fed CT-supplemented concentrate.

In lambs, the incorporation of 25 g/kg DM of grape seed extract in a high-forage diet supplemented or not with a blend of vegetable oils (sunflower and linseed oils, 1:2 (*v/v*)) reduced the MDA levels in meat stored for 3 and 7 days at 4 °C and subjected to lipid oxidation induction [59]. Consistently, Mu et al. [86] reported that the MDA content decreased linearly in lamb meat with increasing levels of grape seed extract (0, 20, 30 and 40 mg/kg body weight/day) in the high-concentrate diet. Furthermore, the increasing levels of grape seed extract in the diet also resulted in a linear increase of the total antioxidant capacity and activity of the CAT, SOD and GPx4 in muscle [86]. Improvement of the total antioxidant capacity, evaluated in plasma by TEAC assay, was also reported by Gladine et al. [87] when grape seed extract was infused directly into rumen. Conversely, the plasma antioxidant activity (FRAP assay) of dairy goats and sheep was not affected by concentrate supplementation with grape seed extract (74 g/kg DM) [88]. Feeding lambs with concentrate diet supplemented with 50 mg/kg DM of grape seed extract also did not affect the MDA and metmyoglobin contents in meat packaged under high O_2_ atmosphere (O_2_:CO_2_, 80:20) and stored at 4 °C for 14 days [52]. Contents of MDA and protein carbonyl in PUFA *n*-3 enriched meat stored in a modified atmosphere (O_2_:CO_2_, 70:30) over 12 days at 4 °C was also not affected by supplementation of lamb diets with 900 mg of red wine extract per kg of concentrate [89].

The efficacy of dietary supplementation with mimosa (*Acacia mearnsii*) CT extract to protect lamb meat against lipid oxidation was tested in two studies, where 40 g/kg of mimosa extract was included in concentrate [90,91]. In both experiments the supplementation of diet with mimosa extract did not affect the MDA levels in raw meat stored at 4 °C over 7 days [90,91], not even in meats subjected to pro-oxidant conditions (cooking and meat homogenate with pro-oxidant catalysts) [91]. However, the effect of mimosa CT extract on oxidation of myoglobin in meat was contradictory: one of these works reported the reduction of metmyoglobin formation [90], while the other showed that supplementation of diets with mimosa CT extract did not affect the myoglobin oxidation [91]. Lipid oxidation in salted and sun-dried meat from bulls fed an oil-supplemented (42.5 g/kg DM of soybean oil) diet composed of 40:60 forage and concentrate was also not affected by increasing levels of mimosa extract in the diets (10, 30 and 50 g/kg DM) [92]. Similar results were also found by Staerfl et al. [93], who reported that supplementation of maize silage with concentrate containing 141 g/kg of mimosa extract did not improved the oxidative stability of perirenal fat of bulls. More recently, Avilla et al. [94] supplemented the diets of lactating dairy cows with increasing levels of *Acacia mearnsii* CT extract (6.12, 12.25; 18.42 and 24.58 g/kg DM) and found no differences in the MDA milk levels. However, they found increasing diene conjugates concentration, which indicates a greater lipid peroxidation, with increasing CT levels.

Although the results point to the effectiveness of CT extracts in improving the overall antioxidant status of ruminants and the oxidative stability of their products, the results are controversial, and for some CT extracts, antioxidant effect in ruminants have not yet been verified, as reported for the mimosa extracts. The inconsistent antioxidant effect in ruminants of the various CT extracts may be related to the chemical structure of CTs, once the CT structure differs markedly according to its origin [22,23]. Moreover, several other factors can contribute to inconsistent antioxidant effect of CT extracts, such as CT concentration in the diets, composition of basal diet, presence of pro- and antioxidant compounds, or other uncontrolled factors as environmental temperature and stress-inducing situations, that might create a different balance between antioxidant and pro-oxidant agents. In addition, the interpretation of results is hampered by the diversity of analytical techniques and standards used to quantify the CTs, and in some studies, such information is not displayed. The CT content in sources and diets has been quantified through butanol-HCl [53,54,56,59,60,61,65,66] and vanillin [71,72] assays and using different standards, such as cyanidin chloride, leucocyanidin, catechin, tannic acid or CT purified from the vegetal material under study (see footnotes of Table 1 and Table 2). Some studies only reported the total tannins content, or the CT levels supplied by the manufacturer without identification of the procedures used for CT quantification, and in others, the CT levels in diets are not reported. The lack of information on the CT contents and the non-standardization of methodologies and standards used for quantification make comparisons between studies difficult. 

If the occurrence of antioxidant compounds other than CTs can contribute to the antioxidant effect when CT-rich plants and by-products are used, the improvement in antioxidant status and oxidative stability observed by the inclusion of the CT extracts in principle are directly related to CT action. However, the mechanisms by which CTs are able to exert an antioxidant effect in ruminants are unclear. So, elucidation of the CT antioxidant mechanisms is a relevant topic that might be useful to understand the controversial results on the antioxidant effect of dietary CTs in ruminants, and to design effective antioxidant strategies for these species based on incorporation of the CT sources in their diets. 

## 4. Condensed Tannins’ Antioxidant Action Mechanisms 

As with other polyphenols, the antioxidant function of CTs involves diverse mechanisms, such as scavenging of free radicals, transition metals chelation and the inhibition of pro-oxidative enzymes [25]. Moreover, antioxidant synergies between exogenous phenolics and endogenous antioxidants have been reported, and it is known that CTs can interfere with the metabolism of other antioxidant compounds [95]. The antioxidant activity of CTs is well established in in vitro models, as well as when directly applied to food products. However, the mechanisms by which CTs can induce antioxidant activity in vivo are still unclear. Unlike other phenolic compounds with low molecular weight, the polymeric nature and high structural complexity of CTs limit their absorption into circulation and the direct antioxidant effect in living animal and post-mortem, making it more difficult to explain the mechanisms by which CTs has antioxidant effect in vivo. Several action mechanisms, involving direct and indirect mechanisms, seem to be possible, such as: (i) absorption of CTs or metabolites allowing direct action in animal body and tissues; (ii) antioxidant action in the gastrointestinal tract (GIT); and (iii) interaction with other antioxidant compounds and endogenous antioxidant system. 

The presence of the CTs in circulation and deposition in tissues would allow a direct antioxidant action. However, the bioavailability (usually defined as the concentration of a given compound or its metabolites that reaches the systemic circulation [96]) of CTs depends highly on the degree of polymerization and molecular weight [22,23]. Condensed tannins are characterized by their polymeric nature and high molecular weight, which limit their bioavailability once only monomers and oligomers up to tetramers can be absorbed and transported via circulation to various organs [23,97,98]. Moreover, between monomers and oligomers that can be absorbed, there are differences in absorption rates, depending on the molecular weight, chemical structure and stereochemical configuration [23,24,97]. The flavan-3-ol monomers show different absorption rates according to chemical structure and stereochemical configuration, decreasing the absorption rate from (−)−epicatechin > (+) − epicatechin = (+) − catechin > (−) − catechin [99]. In addition, the absorption rates of CT dimers, trimers and tetramers decrease with their increasing molecular size and number of hydrophilic hydroxyl groups [97]. Although the CT chemistry varies between plant sources, generally it is mainly composed of oligomers and polymers, and low molecular weight CTs are present in low concentrations [22,23]. So, the contribution of native CT monomers and small oligomers to generate antioxidant effects through their absorption may be limited. 

The degradation of CTs into compounds with a lower degree of polymerization or even to monomers throughout the GIT would allow greater availability of absorbable compounds, but the CT metabolization in the GIT is still poorly understood. Despite the conflicting results, several reports have shown that CTs are not inert during transit through the GIT, instead they are subject to extensive biotransformation processes [23,98,100,101]. Along the GIT, there are conditions like gastric acidity and microorganisms that could promote CT changes. However, CTs are characterized by their high resistance to degradation induced by acid conditions and by most microorganisms [102], which can limit the CT transformation in the GIT. Results on CT degradation under acidic conditions are inconsistent. Degradation of procyanidin oligomers from cocoa and apple with the production of oligomers with a lower degree of polymerization and monomers was observed after in vitro incubations under acid conditions [103,104,105]. On the other hand, simulating the gastric conditions, Li et al. [106] observed only a slight decrease in the degree of polymerization of CTs from *Choerospondias axillaris* peels after incubation. Gültekin-Ozgüven et al. [105] reported that monomers and dimers from cocoa remained stable under acidic conditions. In humans, it was also demonstrated that cocoa CT remained quite stable during in vitro gastric digestion [107]. So, due to their high resistance to acidic conditions and low absorption in the small intestine, it is widely accepted that most of the CTs reaches the colon intact, where they are metabolized through the action of the intestinal microbiota with the production of various low molecular weight compounds that can be absorbed into the circulation [97,102,108]. The transformation of CTs by gut microbiota has been reviewed, with a wide variety of compounds reported to be generated, mainly aromatic acids and valerolactones, which can also contribute to the health effects of CTs [23,97,98,102,108]. Although CTs might undergo extensive metabolization during transit through the GIT, the presence of CT monomers, oligomers, and polymers in feces of animals that received CT sources [109,110,111] suggests that their biotransformation is incomplete [110]. 

The studies on the fate of CTs in the GIT have been performed mainly using in vitro models and in humans and animals such as rats and pigs [23,108], while in ruminants the literature available on CT metabolism throughout GIT is scarce. Recently, Quijada et al. [112] reported a large disappearance of CTs from sainfoin and hazelnut skin between feed and feces (61–85%), suggesting that CTs may be structurally modified, degraded, or absorbed along the GIT. Consistently, the recovery of CTs in feces from sainfoin-fed cows was less than would be expected if the CTs remained inert throughout the GIT [40]. Moreover, a reduction of the mean degree of polymerization (mDP) of CTs in the digestive tract was observed in these cows fed sainfoin [40]. Recently, Girard et al. [37] also reported that only 46% and 78% of CTs were recovered in the large intestine of the lambs fed sainfoin or birdsfoot trefoil silages, respectively. In both lambs fed sainfoin or birdsfoot trefoil silages, soluble CTs were detected in the digesta when the Acetone-HCl-butanol assay was used, but a large part of soluble PC and PD were not detected by UPLC-MS/MS analysis, suggesting the occurrence of chemical transformations of CTs in the digestive tract in compounds that could not be detected by the specific MS/MS method for analysis of PC and PD. Using ^14^C-labeled CTs, a substantial disappearance of CTs from sheep and goat gastrointestinal tracts was also identified [113,114]. Gladine et al. [87] reported the presence of epicatechins in the plasma of sheep that received grape peel and skin extract directly into the rumen. Considering the minor proportion of monomeric compounds in grape peel and skin extract, Gladine et al. [87] associated the presence of epicatechins in the plasma of sheep with the possible biodegradation of polymeric CTs by the ruminal microorganisms. Conversely, Makkar et al. [115,116] could not demonstrate the degradation of CTs by rumen microorganisms. However, it could be hypothesized that CTs can also be metabolized in the rumen and in the intestine of ruminants, similarly to what happens in the colon of humans [37], and further studies using advanced techniques of CT analysis can help to elucidate the fate of CTs in the GIT of ruminants. 

Condensed tannins are known for their ability to form complexes with various types of molecules, primarily with proteins and to a lesser extension with polysaccharides, nucleic acids and metal ions [117]. These complexes are generally unstable depending on numerous factors, including pH, and the pH variation along the ruminants GIT defines the behavior of CTs. Condensed tannins form stable complexes with proteins under the rumen pH conditions (pH 5.5 to 7.0), releasing under the acid conditions of the abomasum (pH 2.5 to 3.5), and in alkaline conditions of the distal small intestine (pH ≈ 7.5) [118,119]. So, the GIT conditions can conditionate the accessibility to CTs and, therefore, their reactivity and bioavailability. 

Despite the conflicting results on CT metabolization and absorption, increasing evidence supports that CTs undergo transformation along the GIT. The discrepancy in these results can be attributed to several factors, such as type of CT (degree of polymerization, chemical structure), possible interactions with food/feed matrix [23], and animal species. Furthermore, methodologies that have been used over time to assess the concentration and composition CTs and their metabolites have different sensitivities, which may also help to explain the conflicting results. 

Several studies reported an increase of the total phenolic content, determined using Folin–Ciocalteau reagent, in plasma, muscle and milk of ruminants fed diets contain CTs (shown in Table 1 and Table 2), which could suggest a possible transfer of dietary CTs to plasma, muscle and milk. However, the Folin–Ciocalteau reagent is not specific to phenolic compounds and can react with other reducing agents [120], so these results should be interpreted with caution. Presence of CT monomers or small oligomers in plasma, muscle and milk of ruminants fed CT sources only was reported by Gladine et al. [87], who identified epicatechins in the plasma of sheep fed supplements with grape peel and skin extract. Conversely, no phenolic compounds were detected in liver and plasma of lambs supplemented with quebracho CT extract [84]. The level of catechins in milk also did not change when increasing levels of *Acacia farnesiana* were added to goats’ diets [64].

Although the CTs in intact form or their metabolites can be absorbed and transported to organs by blood inducing beneficial effects, the great proportion of CTs and their metabolites that are excreted in feces is an indicator of their poor bioavailability [23]. So, the contribution of dietary CTs to animal antioxidant status and oxidative stability of products through absorption is probably very limited, although it cannot be excluded. Other action mechanisms can be involved in the antioxidant activity of CTs in vivo.

The gastrointestinal tract has been proposed as the main site of CTs’ action, where they and/or their metabolites can directly exert several biological activities, as antioxidant activity. In the intestine, CTs can scavenge free radicals, chelate metals, and reduce lipid peroxidation and the production of lipid oxidation derivatives and toxic compounds, which could result in improvements of the animal’s overall antioxidant status [121,122]. Moreover, several studies have shown that CTs are also able to protect other antioxidant molecules from oxidation, such as vitamins. This would increase the amounts of antioxidant compounds along the GIT, contributing to enhance the animal’s antioxidant status [95]. Yamamoto et al. [123] reported the increase of vitamin E levels in the large intestine mucosa of rats fed green tea catechins. The effect of CTs on vitamin E levels does not appear to be limited to GIT alone and increases in vitamin E concentrations have been reported in tissues from animals fed CT sources. The inclusion of grape extract in rat diets increased the vitamin E content by 24% in the liver [124]. Furthermore, it has been reported that several phenolic compounds, including catechins and epicatechins, increase the vitamin E levels in the plasma and liver of rats [125]. Inclusion of 200 g/kg DM of *Cistus ladanifer* in lamb diets also resulted in increased levels of α-tocopherol in muscle [61]. Although *Cistus ladanifer* can be a source of vitamin E, other mechanisms that may be responsible for these higher levels of α-tocopherol in muscle cannot be excluded. Various mechanisms by which phenolic compounds might induce such an increase have been hypothesized, such as by protecting vitamin E against oxidation, restoring the vitamin from oxidation, and inhibition of metabolism and enhancement of absorption [126]. 

Moreover, CTs are also able to protect other antioxidant compounds in addition to vitamin E, such as ascorbic acid [95]. Iglesias et al. [95] demonstrated the strong ability of grape CT to repair oxidized α-tocopherol and to delay the ascorbic acid depletion in muscle tissues of fish. Regeneration of α-tocopherol from tocoperoxyl radical was also observed by Facino et al. [127] by the addition of grape procyanidins to phosphatidylcholine liposomes and red blood cells. So, the interaction with other antioxidant compounds in GIT, increasing their availability for absorption or to exert antioxidant activity in GIT, or even the interaction of CTs that can be absorbed with other antioxidant compounds in circulation or deposited in tissues, are possible mechanisms by which CTs can exert antioxidant activity indirectly. 

Moreover, it has also been shown that CTs’ beneficial effects may be related to an ability to modulate the cell signaling pathways, thus affecting the expression of specific genes [22]. Supplementation of sheep diets with grape skin increases the gene expression of SOD in plasma [128]. More recently, Mu et al. [86] reported increased GPx4 and SOD mRNA abundance in *longissimus dorsi* muscle from lambs fed on grape seed CT. Higher antioxidant enzymes gene expression was also observed in human hepatoblastoma cells incubated with grape seed procyanidins [129]. Consistently, increased activity of antioxidant enzymes was observed in animals fed sources of CT, as presented in previous Section 3.1 and Section 3.2. Increased transcription factor Nrf2 (nuclear factor erythroid 2–related factor 2) was observed in the muscle of lambs fed diets containing wine grape pomace [49] or grape seed CT extract [86]. Nrf2 is a major regulator of antioxidant protective genes, regulating the expression of proteins involved in the antioxidant defense system [130]. The Nrf2 pathway is activated by oxidative stress (excessive levels of ROS), but also by the presence of electrophilic compounds [130,131]. It is reported that some polyphenolic compounds are able to induce endogenous antioxidant defense mechanisms by modulating Nrf2 [130,131,132,133,134]. Among many other phenolic compounds, epicatechin and catechin have already been described as capable of activating the Nrf2 pathway [133]. Despite the poor bioavailability of CTs, the increased expression and activity of antioxidant enzymes and transcription factor Nrf2 observed in ruminants fed CT sources suggest that activation of cell signaling pathways may be another mechanism by which CTs can improve the antioxidant response in living animals.

## 5. Conclusions

The results showed that inclusion of CT-plants and plant extracts in ruminant diets can improve the animal antioxidant status and produce edible products with better oxidative stability. However, the antioxidant effect of dietary CT sources in ruminants has not been proven in all works, which can be due to several factors, such as CT chemical structure and concentration in the diets, composition of basal diet, presence of pro- and antioxidant compounds, or other uncontrolled factors that might create different balance between antioxidant and pro-oxidant agents.

Evidence supports that the antioxidant effects of CTs, which are observed both in living animals and *post-mortem* by preventing oxidative damage in products, may be due to multiple mechanisms of action, which could occur in an integrated and synergic way. The direct antioxidant effect, which implies the absorption of CTs or their metabolites and presence in circulation and tissues, probably is not the most relevant CT antioxidant mechanism considering the poor bioavailability of CTs. However, this mechanism cannot be excluded and can contribute to overall antioxidant status when CT sources contain or originate higher levels of absorbable CTs. The antioxidant action of CTs in the GIT is the most consensual method, which can also occur in different ways, reducing oxidation reactions and protecting other antioxidant compounds. Interaction with other enzymatic and non-enzymatic components of the antioxidant system seems to be another possibility by which CTs can exert antioxidant activity. In addition to other factors, the chemical structure of CTs seems to be an important factor in determining the antioxidant action mechanisms of CTs in vivo, so the way CTs exert antioxidant activity and its effectiveness probably depend on the CT’s origin. However, the chemical structure of several CT sources used in animal nutrition is poorly characterized, and their characterization should be improved. In addition to better knowledge of the chemistry of each CT source, it is important to carry out further studies in ruminants to understand the action mechanism by which each CT source exerts antioxidant activity in living animals and in their products, which will allow design of effective antioxidant strategies based on the application of CT sources in diets.

## Figures and Tables

**Table 1:** Antioxidant effect of utilization of condensed tannin-rich plants and agro-industrial by-products in ruminant diets.

Animal	Source		CT Level in Diets (g/kg DM)	Basal Diet	Sample	Effect	References
	Plant	Level in Diet (g/kg DM)					
Lamb	Grape pomace	50 and 100 ^1^	-	Corn, soybean meal, wheat bran, oil cake of flax seed, naked oat straw and potato rattan	muscle and testes	↓ROS and MDA levels; ↑Total antioxidant capacity; ↑ activity of GPx4 and SOD in muscle and testes; ↑activity of CAT in testes	[49,50]
Lamb	Grape pomace	51.7 and 103	2.21 and 4.41	Forage:concentrate (40:60) supplemented with 2.7% of linseed oil supplied to dams	muscle	↓MDA levels and MMb % in suckling lambs	[51]
Lamb	Grape pomace	50	2.23 ^4^	Barley straw and concentrate ad libitum; grape pomace included in concentrate	muscle	=MDA levels and MMb %	[52]
Lamb	Grape pomace	50, 100, 150 and 200 ^2^	2.7; 3.5; 4.9 and 6.0 ^5^ 0.8; 1.2; 1.8 and 2.6 ^6^	Concentrate: lucerne meal (80:20)	muscle	↑ FRAP values from day 1 to day 3 in the diets with 150 and 200 g/kg grape pomace; ↓ MDA levels from day 5 onward in the diets with 200 g/kg grape pomace; ↓ carbonyl content at days 5 and 7 of storage	[53]
Steers	Grape pomace	150 ^2^	50.7 ^5^ 24.1 ^7^	Wheat straw: concentrate: lucerne (9:80:10)	muscle	↑ FRAP values; ↓ MDA and carbonyl levels	[54]
Cow	Grape pomace silage	50, 75 and 100	-	Silage: concentrate (60:40); grape pomace silage replace partially the corn silage in forage	milk	↑ reducing power; =production of conjugated diene hydroperoxides	[55]
Lamb	Carob pulp	240 and 350 ^2^	3.4 and 4.5 ^8^	Concentrate: dehydrated lucerne (80:20); carob pulp replaces partially barley of the concentrate	muscle	=MDA levels, free thiol and carbonyl levels and MMb %	[56]
Goat	Pine bark	-	130 ^4^	Rye grass pasture supplemented with mixture of pine bark with molasses (6% *w/w*) and alfalfa (5% *w/w*) containing 130 g/kg DM of CT	muscle	=MDA levels	[57]
Goat	Peanut skin	250; 500 and 750^2^	39; 78 and 117 ^2^	Concentrate (cracked corn, soybean meal, soy hull and molasses) and peanut skin	muscle	=MDA levels in diets with 500 and 750 g/kg peanut skin	[58]
Lamb	*Cistus ladanifer* L.	250	20.9 ^9^	Dehydrated lucerne supplemented with 0 or 6% of a blend of sunflower and linseed oils (1:2, *v/v*)	muscle	↓ MDA levels after lipid oxidation induction	[59]
Lamb	*Cistus ladanifer* L.	50, 100 and 200	2.7; 6.9 and 15.6 ^9^	Concentrate: dehydrated lucerne (50:50); supplemented with 0, 4 and 8% of a blend of soybean and linseed oils (1:2, *v/v*); *Cistus ladanifer* replaces partially the forage	muscle	↓ MDA levels; ↓ MDA levels after lipid oxidation induction; ↑ α-tocopherol content; =total phenolic content, FRAP and TEAC values	[60,61]
Lamb	*Cistus ladanifer* L.	150	3.5–5.6 ^9^	Concentrate: dehydrated lucerne (50:50); supplemented with 5–6% of soybean oil; *Cistus ladanifer* replaces partially the forage	muscle	=MDA levels	[62]
Goat	*Larrean divaricata*	125 ^2^	1.74	Alfalfa hay, corn and soybean meal	muscle	=DPPH values; =total phenolic content; ↓ MDA levels in meat stored at 4 °C over 6 days, at 26 °C for 6 h and at −18 °C for 30 days	[63]
	*Acacia aroma*	125 ^2^	5.63				
Goat	*Acacia farnesiana*	100, 200 and 300	-	Lucerne hay and concentrate	milk	↑ total phenolic content; =catechin concentration; ↑ ORAC and FRAP values	[64]
Cow	*Ficus infectoria*	119 ^2^	15^4^	Rice straw, maize green and concentrate (maize, mustard cake and rice bran); *Ficus infectoria* leaves included in concentrate replacing rice bran	erythrocytes	↑ SOD and CAT activity; ↑ GSH levels; ↓ MDA levels; ↑ total thiol levels	[65]
Lamb	*Ficus infectoria*	106, 159 and 212 ^2^	10; 15 and 20 ^4^	Wheat straw, green fodder, and concentrate; *Ficus infectoria* leaves replacing partially the wheat bran of the concentrate	erythrocytes	↑ SOD and CAT activity; ↑GSH levels; ↓ MDA levels; ↑ total thiol and protein thiol levels	[66]
Lamb	*Ficus infectoria* and *Psidium guajava* (70:30)	96; 144 and 192 ^3^	10; 15 and 20 ^4^	Wheat straw, oat hay and concentrate (maize, wheat bran, deoiled soybean meal); leaf meal mixture replace the concentrate	erythrocytes	↑ SOD, GPx and CAT activity; ↑ GSH and GST levels; ↑ total thiol and protein thiol levels; =MDA levels	[67]
Goat	Oak (*Quercus leucotrichophora*)	-	33.5 ^4^	Concentrate: oak leaves as roughage	erythrocytes	↑ SOD and CAT activity; ↑ GSH levels	[68]
Lamb	Sainfoin (*Onobrychis viccifolia*)	-	5.59–6.71 ^4,8^	Silage mixture of timothy and sainfoin (50:50) *ad libitum*, straw (60–80g/d) and barley (229 g/d)	muscle	=MDA levels in raw meat; ↓ MDA levels under pro-oxidant conditions (cooking and incubation with pro-oxidant catalysts)	[69]
Lamb	Sainfoin (*Onobrychis viccifolia*)	-	21.9 ^10^	Sainfoin pasture supplied to dams	muscle	↓ MDA levels suckling lambs	[70]
Steers	High-tannin sorghum	383–765	17.3–34.6 ^11^	Silage:concentrate (10:90); high-tannin sorghum replace partially the corn of the concentrate	muscle	↓ MDA levels in vacuum-packaged beef; ↑ MDA levels in displayed beef over 6, 10 and 15 days; =SOD, CAT and GPx activity	[71]
Lamb	Sorghum grain	100; 200 and 400	8.2; 16.4 and 24.5 ^11^	Forage (*Aneurolepidium Chinense* hay and alfalfa hay):concentrate (corn grain and soybean meal) (42:58). Stepwise replacement of corn grain by sorghum grain	muscle	=MDA levels; ↑ tannin levels	[72]

^1^ Expressed on air-dry matter basis; ^2^ expressed on an as-fed basis; ^3^ calculated according to CT levels of leaf meal mixture; ^4^ CT levels in CT source or in supplement mixture; ^5^ total tannins expressed as g gallic acid equivalent/kg DM; ^6^ CT expressed as g cyanidin chloride equivalates/kg DM; ^7^ CT expressed as % leucocyanidin equivalates; ^8^ total tannins expressed as g tannic acid equivalents/kg DM; ^9^ CT quantified using *Cistus ladanifer* purified CT as standards; ^10^ CT expressed as g cyanidin equivalent/kg DM; ^11^ condensed tannins expressed as g catechin equivalents/kg DM; DM—dry matter; CT—Condensed tannins; CAT—catalase; DPPH—2,2-Diphenyl-1-picrylhydrazyl assay; FRAP—reducing ability assay; GPx—glutathione peroxidase; GSH—glutathione; MDA—malondialdehyde; MMb—metmyoglobin; ROS—reactive oxygen species; SOD—superoxide dismutase; TEAC—radical scavenging ability,↑—increase; ↓—reduction.

**Table 2:** Antioxidant effect of utilization of condensed tannin extracts in ruminant diets.

Animal	Source			CT Level (g/kg DM)	Basal Diet	Sample	Effect	References
	Plant	CT Levels of Extract (g/kg DM)	CT Extract Levels (g/kg DM)					
Lamb	Quebracho	-	50 ^1^	3.7 ^6^	Concentrate (corn, soybean meal, wheat and barley) + straw *ad libitum.* Quebracho extract included in concentrate	muscle	=MDA and MMb levels	[70]
Lamb	Quebracho	750 ^1^	100 ^1^	75 ^1,5^	Dietary treatments supplied to dams. Pasture vs. forage diets supplemented with concentrate. Quebracho extract included in concentrate	muscle	↓ MDA levels; ↑ α-tocopherol levels in muscle of suckling lambs; =MMb levels	[80]
Lamb	Quebracho (*Schinopsis lorentzii*)	-	89	40.4 ^2^	High-concentrate diet (barley and soyabean meal) and lucerne hay; quebracho extract included in concentrate and forage mixture	muscle	=MDA levels; ↑ total phenols levels; ↑ FRAP and TEAC values; ↓ MMb %	[82,83]
Lamb	Quebracho (*Schinopsis lorentzii*)	-	95.7	64	High-concentrate diet (barley and soyabean meal) and lucerne hay; quebracho extract included in concentrate and forage mixture	liver	=total phenolic content; ↑ FRAP values in raw samples; =total phenolic content and FRAP values in SPE samples	[84]
						plasma	↑ total phenolic content and FRAP values in raw samples; =total phenolic content and FRAP values in SPE samples	
Sheep	Quebracho (*Schinopsis lorentzii*)	456 ^2^	52.8 ^5^	16 g/kg DM intake	250 g Chopped grass hay + 800 g concentrate/day	plasma	=MDA levels	[85]
Sheep	Quebracho (*Aspidosperma quebracho*)	-	80 ^1^	-	Dried beet pulp supplemented with 2% of vegetable oil	muscle	=MDA levels	[81]
Lamb	Grape seed (*Vinis vitifera*)	950	25	14.1 ^7^	Dehydrated lucerne supplemented with 0 or 6% of a mixture of sunflower and linseed oils (1:2, *v/v*)	muscle	↓ MDA levels after lipid oxidation induction	[59]
Sheep	Grape peel and seed (*Vinis vitifera*)	>800	10% DM intake	-	Concentrate (barley, beet pulp, soybean meal, molasses): meadow hay (30:70)	plasma	↑ TEAC values; ↑ the length of the lag phase of conjugated dienes generation; presence of five different phenolic compounds, including epicatechin	[87]
Lamb	Grape seed	-	10, 20 and 40 mg/kg BW/day	-	Total mixed feed with concentrate (corn, soybean meal, cottonseed meal, wheat bran): forage (corn and millet straw) (70:30)	muscle	↑ total antioxidant capacity, ↑ activity of CAT, SOD and GPx4; ↓ MDA levels	[86]
Lamb	Grape seed	413	50 mg extract/kg DM ^5^	-	Barley straw and concentrate (barley, soya and molasses) ad libitum, grape seed extract included in concentrate	muscle	=MDA levels and MMb %;	[52]
Sheep Goat	Grape seed	-	74 ^5^	7.3–7.5 ^4^	Forage:concentrate:dried sugar beet pulp (51:46:3), grape seed included in concentrate	milk plasma	=FRAP values in plasma; ↑ total phenol concentration in plasma and milk	[88]
Lamb	Red wine extract	-	900 mg extract/kg feed ^5^	-	Barley + concentrate (corn meal, barley, wheat, soybean meal, sunflower meal) supplemented with extruded linseed and deodorized fish oil; red wine extract included in concentrate	muscle	=MDA and protein carbonyl levels; =total phenols content	[89]
Lamb	Mimosa (*Acacia mearnsii*)	^-^	40 ^1^	22.3 ^2^	Concentrate (barley, wheat bran, soybean meal, molasses): dehydrated lucerne (85:15)	muscle	=MDA levels; ↓ MMb %	[90]
Lamb	Mimosa (*Acacia mearnsii*)	881 ^3^	40 ^1^	22.3 ^2^	Concentrate (barley, wheat bran, soybean meal, molasses): dehydrated lucerne (85:15)	muscle	=MDA levels in raw and cooked meat and in meat homogenates with Fe3+/Asc; did not after color stability	[91]
Bull	Mimosa (*Acacia mearnsii*)	720 ^3^	10, 30 and 50	-	Concentrate (corn, soybean meal, soybean oil (4.3%)): Tifton-85 hay (60:40)	muscle	=MDA levels in salted and sun-dried meat	[92]
Bull	Mimosa (*Acacia mearnsii*)	700	141 ^1,5^	-	Maize silage:concentrate; mimosa extract included in concentrate	perirenal fat	=oxidative stability evaluated by rancimat test	[93]
Cow	Mimosa (*Acacia mearnsii*)	805 ^1^	6.1; 12.2; 18.4 and 24.6	5; 10; 15 and 20	Concentrate: forage (80:20)	milk	=MDA levels and reducing power; ↑ diene conjugates concentration	[94]

^1^ As fed basis; ^2^ total tannins expressed as g of tannic acid equivalents/kg DM, ^3^ total tannins; ^4^ tannins expressed as g of tannic acid equivalents/kg DM; ^5^ condensed tannin levels in CT source or supplement mixture; ^6^ condensed tannins expressed as g of cyanidin equivalents/kg DM; ^7^ CT quantified using grape seed purified CT as standards; DM—dry matter; CT—Condensed tannins; CAT—catalase; FRAP—reducing ability assay; GPx—glutathione peroxidase; MDA—malondialdehyde; MMb—metmyoglobin; SPE—solid-phase extraction; SOD—superoxide dismutase; TEAC—radical scavenging ability, ↑— increase; ↓— reduction.
